# Invitation to an open debate on non-human primates for research purposes

**DOI:** 10.1093/nsr/nwz062

**Published:** 2019-05-16

In two separate issues this year, *National Science Review* (NSR) published three research articles that described the use of old world monkeys in gene-editing experiments [1–3]. There has been substantial attention on these studies. Aside from the purely scientific issues, the ethical considerations are inherent in this type of research. Since the intellectual, cultural and religious bases of commentaries vary greatly, we believe that the research community has to confront the ethical issues in a comprehensive and open manner. Criticisms from anonymous sources charging the authors of being ‘reckless’ are not the appropriate way to conduct the debate.

NSR has established a Critique and Debate category that accepts submissions of critiques that challenge recent publications in any reputable journal, including NSR itself. We now invite submissions that address the broad issues pertaining to research, gene editing in particular, on non-human primates. Several issues deserve careful examination:

- Monkeys are already used extensively by pharmaceutical companies and basic or clinical laboratories. They have developed protocols for which ethical codes are well defined. We are therefore soliciting critiques and comments specifically on the ethical issues associated with gene editing in non-human primates. For example, is gene editing ethically more (or less) problematic than that of current standards in the use of many animal model systems?
- More generally, what might be the criteria for using monkeys in experiments? What should be considered unacceptable suffering by the animals? What level of benefits to humans should be a pre-requisite? (Alternatively, some could argue that there should be benefits to the animals themselves and benefits to humans are irrelevant in the ethical debate.) For example, Shi *et al.* [3] address a fundamental question about the evolution of humans vis-à-vis other primates. Since the neotenic development in humans [4] can only be investigated through this type of experiment, is the pure intellectual pursuit justifiable for editing the genomes of monkeys? Can further health benefits to humans be pursued through such experiments?
- In debating the ethical issue on the use of non-human primates, it is crucial to consider the evolution of cognitive capacity and mental state. If the evolution of a sense of individuality and empathy is a main criterion, do we apply the knowledge to all taxa of the same taxonomic rank in the strict cladistics sense? Can we apply findings from the lower taxonomic rank to a higher one? For example, can we extrapolate the knowledge on the old world monkeys to all monkeys, which would include the new world marmosets?
- As one of the authors complained in an interview, there is veiled stereotyping that describes such research as ‘Chinese’. There are even attempts to link the NSR publications to the recent much reviled practice of gene editing on human embryos. Despite (or because of) the cultural sensitivity, NSR welcomes explicit examinations of the cultural, religious and sociological dimensions of non-human primate research.

Manuscripts relevant to this debate should be submitted to the editorial office of NSR. The submitted manuscripts will be published after review by the editorial board in the order of the submission date in the coming NSR issues of 2019.
